# Exploring the self-reported physical fitness and self-rated health, mental health disorders, and body satisfaction among Chinese adolescents: A cross-sectional study

**DOI:** 10.3389/fpsyg.2022.1003231

**Published:** 2022-09-15

**Authors:** Chongyan Shi, Jin Yan, Lei Wang, Hejun Shen

**Affiliations:** ^1^School of Physical Education and Humanity, Nanjing Sport Institute, Nanjing, China; ^2^Centre for Active Living and Learning, University of Newcastle, Callaghan, NSW, Australia; ^3^College of Human and Social Futures, University of Newcastle, Callaghan, NSW, Australia; ^4^School of Physical Education, Shanghai University of Sport, Shanghai, China

**Keywords:** fitness promotion, overall health, depression, anxiety, body, school-aged student

## Abstract

**Background:**

Physical activity (PA) and Physical fitness (PF) have received tremendous attention in the field of physical and mental health. However, limited attention has been given to the associations of self-reported physical fitness with some health-related outcomes. Given the COVID-19 pandemic is still active in many Chinese regions, assessing health-related physical fitness (HRPF) in adolescents using field-based assessment (such as a shuttle run for cardiorespiratory fitness) is unrealistic, therefore, this study was conducted via a self-reported questionnaire.

**Purpose:**

The present cross-sectional study was aimed at delving into the relationship between self-reported physical fitness with self-rated health, depression, anxiety, and body satisfaction in adolescents. Three thousand eight hundred and seven study participants from 12 public schools in South-eastern China were recruited and 2,407 of them provided valid data on variables that this study needed for analysis.

**Materials and methods:**

Study participants were asked to self-report their sociodemographic factors (e.g., sex, grade, age), independence, and outcomes. Generalized linear models were used to explore the associations of self-reported physical fitness (comprising general physical fitness, cardiorespiratory fitness, muscular strength, speed and agility, and flexibility) with depression, anxiety, and body satisfaction. A total of 2,407 children and adolescents with a mean age of 13.82 (±2.1) years were included in the final study analysis.

**Results:**

Higher self-reported levels of general physical fitness and cardiorespiratory fitness were associated with better self-rated health, and body satisfaction but with lower risks of depression and anxiety.

**Conclusion:**

The current study offered evidence on the roles of self-reported physical fitness and health-related outcomes. To facilitate health in children and adolescents, advocating fitness education, and promotion could be a feasible approach.

## Introduction

There is a causal relationship between physical activity (PA) and physical fitness (PF) (Zhang et al., 2022). PA has been considered one of the most significant health-related aspects of the lives of children and adolescents (Bermejo-Cantarero et al., 2021; Ren et al., 2021). It is well-established that regular engaging PA has proven to be a vital method for improving body and tissue functions (D’Isanto et al., 2019; Chen and Yan, 2020; Ren et al., 2021). Evidence demonstrates that regular PA can prevent and treat physical diseases (Eather et al., 2013; Lin and Yan, 2020; Shen et al., 2020), as well as play a crucial role in improving mental health (Stanton et al., 2014; Liu et al., 2022).

It is widely accepted that PF can be evaluated as the comprehensive ability of the body to adapt to the surrounding environment (Ullah et al., 2012; Chen et al., 2022; Shi et al., 2022). The definition endowed by Marques et al. (2017) is that it enables everyone to choose the most suitable form and intensity of exercise for their own needs in all kinds of situations to improve their physical fitness and maintain the best health. The authors summarized fitness as the support of four factors, including the ability to perform physical activities that require speed, endurance, strength, and flexibility (Marques et al., 2017). Physical fitness is divided into health-related and skill-related health (Grier et al., 2017; Smits-Engelsman et al., 2020). The five components that get involved in health-related physical fitness (HRPF) include cardiorespiratory fitness, body composition, muscle strength, muscle endurance, and flexibility (Gu et al., 2016; Kim et al., 2021). There is considerable evidence that increasing HRPF levels among adolescents have health and academic benefits (Cattuzzo et al., 2016). For instance, adolescents with high HRPF levels are only less likely to develop cardiovascular disease later in life (Baumgartner et al., 2020). Adolescents’ cardiopulmonary health has a positive correlation with their academic performance due to its promoting effect on psychological emotion and learning concentration (Bailey et al., 2009; Padilla-Moledo et al., 2020; Liu et al., 2022), and a study by Del Giudice et al. (2021) found that a lack of daily step counts would have higher odds of being hypogonadal. Muscle health, such as strength and endurance, makes adolescents with low-risk obesity more likely to be active in physical activities (Malm et al., 2019; Chen and Yan, 2020; Lin and Yan, 2020; Comeras-Chueca et al., 2021; Gu et al., 2022). Moreover, a random control trial study by Mavilidi et al. (2021) found that PF can significantly improve children and adolescents’ cognitive control, working memory, and subjective vitality in a school setting. Of note, cross-sectional research has revealed that children and youth with higher fitness levels have greater hippocampal volume and memory performance compared with lower-fit children (Chaddock et al., 2010). It can be explained that regular engaging PT could stimulate angiogenesis and neurogenesis in areas of the brain, which ultimately influence cognitive performance (Leahy et al., 2020).

Self-reported physical fitness through questionnaire scale tools such as comprehensive evaluation of adolescent health contains four independent variables, including health, anxiety, as well as body satisfaction (Moliner-Urdiales et al., 2010; Barnes et al., 2020). First and foremost, self-rated health shows a positive correlation with physical fitness, and the self-rated health assessment of adolescents is affected by gender and grade (Vingilis et al., 2002; Warnoff et al., 2016). For example, according to Schrotenboer’s (2018) interview data in 2018, male students were significantly more satisfied with their physical and mental conditions and learning status than that female students, showing the differences in psychological characteristics and content of attention between men and women (Schrotenboer, 2018). They also paid less attention to their health and behavior, and their emotional experiences were less profound than those of their female counterparts. What’s more, depression and anxiety are both manifestations of emotional loss, which can cause great trouble to teenagers’ life and studies (Michl et al., 2013). In terms of these two indicators, teenagers who think their mental state is relatively satisfied generally argue that their physical fitness is at an intermediate or upper level (Mulasi-Pokhriyal and Smith, 2010; Csikszentmihalyi and Hunter, 2014). However, teenagers who rate themselves poorly also have lower health ratings and are more likely to be academically disadvantaged (Suldo and Shaffer, 2008). Thirdly, body satisfaction is often a factor that determines whether teenagers have emotional problems, and it is mainly attributed to their confidence in their appearance and body (Carlson Jones, 2004; Presnell et al., 2004).

Some studies get involved in the degree of interference of external factors on adolescents’ self-health cognition and life satisfaction. Novak et al. (2017) suggested that there was a significant correlation between values and life satisfaction in special fields, but the correlation coefficients were all very low. By contrast, the correlation between self-concept and life satisfaction was higher, implying that the relationship between self-concept and life satisfaction might be closer than that between values input from the external environment and life satisfaction (Brillhart, 2005). At the same time, Galán et al. (2013) took regional and family environment as dependent variables to reveal the overall satisfaction of urban students and the satisfaction score of school and living environment was significantly higher than that of rural students. As a result, it is suggested that schools and communities should make efforts to reinforce adolescent sports activities and health evaluation systems to make sure the establishment of basic life satisfaction for adolescents (Oberle et al., 2011; Padilla-Moledo et al., 2020).

With the COVID-19 pandemic still ongoing in many Chinese areas, assessing HRPF in youth using field-based assessment (such as a shuttle run for cardiorespiratory fitness) is unrealistic. Therefore, finding an alternative to assess HRPF in adolescents is necessary. The International Fitness Scale (IFIS) is an option that can consider adolescents’ HRPF conveniently (Ortega et al., 2011). This study was targeted at further estimating the relationship between self-reported fitness and health-related independent variables to highlight the role of fitness concerning self-rated health, depression, anxiety, and body satisfaction by using a sample of Chinese adolescents.

## Materials and methods

### Study design and participants

This cross-sectional study has been conducted in south-eastern China from March to October 2021. Using convenience sampling, 12 public schools were invited, and these schools were located in four cities in south-eastern China, that is, two high schools, five middle schools, and five elementary schools. For each school, we have randomly selected one or three classes, and a contact person is sent to each school. Once a potential school had expressed interest in this study, the first author emailed, phoned, or zoom called the representative(s) (e.g., the principal and administrative staff), to elaborate on the study requirements and process. The self-report scales were applied at the school by the staff teacher staff or headmaster of the school and they helped to manage and fill out the questionnaire in the form of paper-based. Having completed this step, we have recruited the first group of respondents comprising 3,807 adolescents and children (ages 11–17).

Study participants providing information on variables of interest were included in this study, while those who did not report data on any variables (e.g., independents, outcomes and covariates) of interest that this study needed were excluded from the initial sample. For this study and further analysis, a total of 2,407 study participants were included as they provided valid data on variables this study needed. Moreover, we have informed all the respondents and their guardians and parents that they are completely voluntary to take the survey. The study procedure and protocol have been performed upon approval from the Institutional Review Board (IRB) of the Shanghai University of Sport, and the grant number is 102772021RT071.

### Physical fitness

With the 5-point Likert scale (very bad, bad, fair, good, and excellent), this study used the International Fitness Scale (IFIS) for assessing physical fitness. There are five components in IFIS, which are respectively general fitness, cardiorespiratory fitness, muscle strength, speed and agility, and flexibility. One of the questions used in this study is as follows: What is my general physical condition? (1 = very bad, 2 = bad, 3 = fair, 4 = good, 5 = excellent). The acceptable credibility and validity in teenagers (Ortega et al., 2011) have been reported by the scale. Furthermore, this study recruited 544 Chinese teenagers to attend credibility research. The research results demonstrated the acceptable credibility of IFIS in Chinese children and teenagers (weighted Kappa: 0.42–0.52 for the IFIS component, Cronbach alpha 0.72) (Bao et al., 2022).

### Nine-item patient health questionnaire depression

The Chinese version of the 9-item Patient Health Questionnaire (PHQ-9) was applied to analyse depressive symptoms, including nine items relating to experiences of depressive symptoms in the past 2 weeks. Every item was scored in line with the Likert Scale with four grades, varying from 0 (no scoring) to 3 (almost scoring every day). The total scores ranged from 0 to 27, showing that the higher scores, the more serious depressive symptoms. The severity of depressive symptoms could be divided according to PHQ-9 scores: 20–27 (severe), 15–19 (moderately severe), 10–14 (moderate), 5–9 (mild), and 0–4 (minimal) (Levis et al., 2019). The psychometric properties of PHQ-9 have been tested on Chinese children, showing sufficient validity and reliability (Li et al., 2021).

### Seven-item generalized anxiety disorder scale anxiety

Seven-item Generalized Anxiety Disorder Scale (GAD-7) was used to assess anxiety disorders, including seven items, in which each item’s response had a four-point Likert scale (ranging from 0 to 3) (Maggi et al., 2019). The total scores of GAD-7 ranged from 0 to 21, implying that the higher scores, the higher severity of anxiety. The severity of anxiety could be divided into severe (15–21), moderate (10–14), mild (5–9), and minimal (0–4) (Plummer et al., 2016). The translated GAD-7 has been widely applied among Chinese children and adolescents, showing acceptable validity and reliability (Sun et al., 2021).

### Self-rated health

By asking the following questions, self-rated life satisfaction is measured. Generally, how do you consider your life satisfaction? The possible answers included: “Excellent (5),” “Very good (4),” “Good (4),” “Fair (3),” and “Poor (1)” (Mewes and Giordano, 2017). As for self-rated life satisfaction and its items, sound validity has been documented in previous studies (Zhang et al., 2019; Chai et al., 2020).

### Body satisfaction

The status of body satisfaction is evaluated by asking the question “do you feel satisfied with your body weight of yours” There are five options for the answer ranging from “very satisfied” to “very dissatisfied” (Ren et al., 2018). In the analysis of data, the options of “somewhat dissatisfied” and “very dissatisfied” are combined as one option, that is, “dissatisfaction,” while “very satisfied” and “somewhat dissatisfied” is turned into “satisfaction.” Therefore, the status of body satisfaction has three types, satisfaction, neither and dissatisfaction.

### Demographic characteristics

Age, sex, residence, siblings, living with parents and family affluence were collected as demographic characteristics in the current study. These variables were assessed *via* the following measurement questions: age (continuous), sex (boy or girl), residence (rural, suburban and urban), siblings (yes or no), living with parent (yes or no), and family affluence (0–10 scale).

### Statistical analysis

All analyses were conducted using the Statistical Package for the Social Sciences v.26 (SPSS Inc., Chicago, IL, USA), and the alpha level was set at *p* < 0.05. Descriptive statistics (mean/standard deviation and percentage) were used to report on the basic characteristics of the study sample. Mean value with standard deviation was a continuous variable (e.g., age), whilst percentage was a categorical variable (e.g., sex, residence). Four regression models were established, including self-reported physical fitness and self-rated health, self-reported physical fitness and body satisfaction, self-reported physical fitness and depression, and self-reported physical fitness and anxiety. In each model, the indicators of self-reported physical fitness were entered into the model concurrently while controlling for covariates. A generalized linear model with ordinal logistic regression was used to achieve the association estimation above noted.

## Results

Table 1 demonstrates that these samples were concentrated at the age of 13.9 (± 2.1), and were evenly distributed between boys and girls. Boys accounted for 5.4% more than girls, with 1,236 males and 1,108 girls. More information on the study participants can be found in Table 1.

**TABLE 1:** Sample characteristics of this study.

		n/Mean	%/SD
Gender	Boy	1,236	52.7
	Girl	1,108	47.3
Residence	Rural	266	11.3
	Suburban	514	21.9
	Urban	1,564	66.7
Siblings	Yes	1,155	49.3
	No	1,189	50.7
Live with parent	Yes	1,972	84.1
	No	372	15.9

Table 2 shows that adolescents are generally in their health assessment and choose “Average.” This answer accounted for the highest proportion in the five self-assessments of physical fitness, cardiovascular health, muscle strength, speed and agility, and flexibility. More than 50% of teenagers chose “Average” for Physical Fitness and Muscle Strength. Compared with the other four factors, adolescents had the most negative evaluation of flexibility, 161 of them thought very poorly, and more than 1/5 of them thought poor, 517 of them. In addition, in the sample, the evaluation of comprehensive Physical Fitness was more positive, except for average, accounting for 52.5%, and those who thought “good” and “very good” accounted for 31.9%.

**TABLE 2:** The proportion of different categories of self-reported physical fitness.

		*n*	%
Self-reported physical fitness	Very poor	75	3.2
	Poor	290	12.4
	Average	1,231	52.5
	Good	559	23.8
	Very good	189	8.1
Self-reported cardiorespiratory fitness	Very poor	88	3.8
	Poor	335	14.3
	Average	1,113	47.5
	Good	599	25.6
	Very good	209	8.9
Self-reported muscle strength	Very poor	83	3.5
	Poor	372	15.9
	Average	1,207	51.5
	Good	534	22.8
	Very good	148	6.3
Self-reported speed and agility	Very poor	61	2.6
	Poor	300	12.8
	Average	1,095	46.7
	Good	646	27.6
	Very good	242	10.3
Self-reported flexibility	Very poor	161	6.9
	Poor	517	22.1
	Average	1,009	43.0
	Good	477	20.3
	Very good	180	7.7

Table 3 displays the emotion and satisfaction of adolescents. In terms of Depression severity and anxiety severity, they show a similar trend, which is more than half of the teenagers thought they were normal. However, in terms of body satisfaction, the proportion of Dissatisfied and Neither was close, both roughly 30%. Concerning self-rated health, most the youth choose poor health, the detailed information can be found in Table 3.

**TABLE 3:** The proportion of different categories of outcomes of this study.

		*n*	%
Depression severity	Normal	1,337	57.5
	Mild	627	26.9
	Moderate	194	8.3
	Moderately severe	90	3.9
	Severe	79	3.4
Anxiety severity	Normal	1,595	68.5
	Mild	466	20.0
	Moderate	146	6.3
	Severe	120	5.2
Body satisfaction	Very dissatisfied	268	11.4
	Dissatisfied	732	31.2
	Neither	768	32.8
	Satisfied	317	13.5
	very satisfied	259	11.0
Self-rated health	Very poor	150	6.4
	Poor	851	36.3
	Average	715	30.5
	Good	356	15.2
	Very good	272	11.6

Table 4 presents that the independent variable analysis of self-rated health was strongly correlated with the result of self-rated physical fitness. In detail, compared with those who reported “very poor” self-rated health, adolescents who reported “very good” were more likely to have a high-level physical fitness ([very good] OR = 14.95, 95% CI: 7.53–29.68; [good] OR = 4.18, 95% CI: 2.35–7.43; [average] OR = 1.90, 95% CI: 1.09–3.29; [poor] OR = 1.34, 95% CI: 0.76–2.39). However, the evaluation of flexibility and speed were not the basis for them to identify high physical fitness, and there was no positive correlation.

**TABLE 4:** The association between self-reported physical fitness and self-rated health.

		OR	95% CI		*P*-value
General physical fitness	Very good	14.95	7.53	29.68	0.000
	Good	4.18	2.35	7.43	0.000
	Average	1.90	1.09	3.29	0.023
	Poor	1.34	0.76	2.39	0.314
	Very poor	REF			
Self-reported cardiorespiratory fitness	Very good	4.34	2.24	8.43	0.000
	Good	4.98	2.80	8.86	0.000
	Average	3.22	1.84	5.62	0.000
	Poor	2.45	1.37	4.37	0.002
	Very poor	REF			
Self-reported muscle strength	Very good	1.84	0.93	3.65	0.080
	Good	1.29	0.73	2.28	0.384
	Average	1.51	0.87	2.64	0.146
	Poor	1.13	0.64	2.01	0.676
	Very poor	REF			
Self-reported speed and agility	Very good	1.00	0.48	2.05	0.990
	Good	1.05	0.54	2.06	0.878
	Average	0.99	0.51	1.91	0.977
	Poor	0.66	0.34	1.29	0.225
	Very poor	REF	.	.	
Self-reported flexibility	Very good	0.76	0.48	1.20	0.238
	Good	1.31	0.91	1.89	0.147
	Average	1.10	0.78	1.55	0.584
	Poor	1.00	0.70	1.43	0.999
	Very poor	REF	.	.	

OR, odds ratio; CI, confidence interval; REF, reference.

Table 5 displays the association between self-reported physical fitness and body satisfaction. Similarly, compared with those who reported “very poor” body satisfaction, adolescents who reported “very good” were more likely to have a high-level physical fitness ([very good] OR = 3.62, 95% CI: 1.89–6.93; [good] OR = 2.41, 95% CI: 1.39–4.18; [average] OR = 1.83, 95% CI: 1.08–3.10; [poor] OR = 1.20, 95% CI: 0.69–2.09). However, youth who rated themselves low on flexibility or muscle strength were also likely to report better body satisfaction.

**TABLE 5:** The association between self-reported physical fitness and body satisfaction.

		OR	95% CI		*P*-value
General physical fitness	Very good	3.62	1.89	6.93	0.000
	Good	2.41	1.39	4.18	0.002
	Average	1.83	1.08	3.10	0.024
	Poor	1.20	0.69	2.09	0.515
	Very poor	REF			
Self-reported cardiorespiratory fitness	Very good	2.55	1.39	4.67	0.003
	Good	2.04	1.19	3.50	0.010
	Average	1.42	0.84	2.39	0.188
	Poor	1.29	0.75	2.23	0.352
	Very poor	REF			
Self-reported muscle strength	Very good	0.46	0.24	0.90	0.022
	Good	0.62	0.36	1.05	0.076
	Average	0.86	0.51	1.44	0.559
	Poor	0.85	0.50	1.45	0.556
	Very poor	REF			
Self-reported speed and agility	Very good	1.38	0.72	2.64	0.334
	Good	0.87	0.48	1.60	0.663
	Average	0.64	0.35	1.16	0.140
	Poor	0.53	0.29	0.96	0.038
	Very poor	REF			
Self-reported flexibility	Very good	1.73	1.10	2.70	0.017
	Good	1.84	1.28	2.64	0.001
	Average	1.68	1.19	2.37	0.003
	Poor	1.88	1.32	2.66	0.000
	Very poor	REF			

OR, odds ratio; CI, confidence interval; REF, reference.

In contrast, Table 6 demonstrates adolescents who rated their higher depression were more likely to have lower physical fitness ([very good] OR = 0.21, 95% CI: 0.10–0.41; [good] OR = 0.29, 95% CI: 0.17–0.51; [average] OR = 0.36, 95% CI: 0.22–0.61; [poor] OR = 0.50, 95% CI: 0.29–0.86), which is showing a strong inverse correlation. Secondly, depression was also inversely correlated with the reported physical fitness scores, but not strongly correlated with cardiopulmonary health. There was no correlation between muscle strength and depression, that is, adolescents with high recognition of their muscle strength also had high levels of depression. Depression was not associated with flexibility, and there were no significant differences in depression among different grades of flexibility, more information can be found in Table 6.

**TABLE 6:** The association between self-reported physical fitness and depression.

		OR	95% CI		*P*-value
General physical fitness	Very good	0.21	0.10	0.41	0.000
	Good	0.29	0.17	0.51	0.000
	Average	0.36	0.22	0.61	0.000
	Poor	0.50	0.29	0.86	0.012
	Very poor	REF			
Self-reported cardiorespiratory fitness	Very good	0.67	0.35	1.30	0.240
	Good	0.75	0.43	1.31	0.316
	Average	0.70	0.41	1.18	0.181
	Poor	0.92	0.53	1.60	0.768
	Very poor	REF			
Self-reported muscle strength	Very good	1.02	0.51	2.03	0.959
	Good	0.78	0.45	1.33	0.357
	Average	0.69	0.41	1.16	0.161
	Poor	0.80	0.46	1.37	0.414
	Very poor	REF			
Self-reported speed and agility	Very good	0.78	0.38	1.60	0.495
	Good	1.23	0.64	2.39	0.533
	Average	1.14	0.60	2.19	0.690
	Poor	1.35	0.70	2.61	0.370
	Very poor	REF			
Self-reported flexibility	Very good	0.66	0.41	1.06	0.083
	Good	0.56	0.38	0.81	0.002
	Average	0.61	0.43	0.86	0.005
	Poor	0.66	0.46	0.94	0.020
	Very poor	REF			

OR, odds ratio; CI, confidence interval; REF, reference.

Table 7 displays that anxiety and depression shows similar trends ([very good] OR = 0.22, 95% CI: 0.11–0.47; [good] OR = 0.26, 95% CI: 0.14–0.46; [average] OR = 0.31, 95% CI: 0.18–0.53; [poor] OR = 0.42, 95% CI: 0.24–0.75). More information can be found in Table 6.

**TABLE 7:** The association between self-reported physical fitness and anxiety.

		OR	95% CI		*P*-value
General physical fitness	Very good	0.22	0.11	0.47	0.000
	Good	0.26	0.14	0.46	0.000
	Average	0.31	0.18	0.53	0.000
	Poor	0.42	0.24	0.75	0.003
	Very poor	REF			
Self-reported cardiorespiratory fitness	Very good	0.49	0.24	0.99	0.047
	Good	0.65	0.36	1.17	0.152
	Average	0.67	0.38	1.17	0.158
	Poor	0.71	0.40	1.28	0.255
	Very poor	REF			
Self-reported muscle strength	Very good	0.94	0.46	1.94	0.877
	Good	0.65	0.37	1.14	0.135
	Average	0.69	0.40	1.18	0.169
	Poor	0.76	0.44	1.34	0.348
	Very poor	REF			
Self-reported speed and agility	Very good	1.47	0.67	3.23	0.337
	Good	1.81	0.88	3.72	0.106
	Average	1.58	0.78	3.21	0.206
	Poor	1.47	0.72	3.01	0.292
	Very poor	REF			
Self-reported flexibility	Very good	0.84	0.51	1.38	0.490
	Good	0.61	0.41	0.90	0.014
	Average	0.64	0.44	0.92	0.017
	Poor	0.65	0.44	0.95	0.026
	Very poor	REF			

## Discussion

The primary of this study was to examine the association between self-reported fitness and health-related independent variables in adolescents (aged 11–17) by using the self-reported questionnaire, owing to the COVID-19 pandemic in many regions in China. Our study highlights the role of physical variables in adopting physical activity during adolescence, which proposes specific measures for communities and schools to improve adolescents’ physical and mental health through the mechanism of influence.

The results demonstrated that cardiorespiratory health, muscle health, and speed agility were all related to the perceived health condition, which is consistent with previous findings (Marques et al., 2017; Redondo-Tébar et al., 2019; Bermejo-Cantarero et al., 2021). The healthier respondents are more likely to evaluate themselves with higher scores on physical condition. Among the four components of evaluating general physical fitness, cardiorespiratory health and muscle strength show a significant relationship with self-reported physical health. However, no significant correlation has been identified between flexibility or speed and agility. This shows that in the evaluation of physical health, greater consideration has been given to cardiorespiratory health and muscle strength than flexibility, speed and agility. The same condition has also been detected in the scale of body satisfaction. Adolescents with lower self-perceived muscle strength and flexibility can also report a higher level of body satisfaction. Based on the above discussion, it can be drawn that adolescents report their overall fitness condition based on their perception of cardiorespiratory function and muscle strength. This result is in line with what Marques et al. (2017) have discovered, whose evidence showed a positive relationship between cardiorespiratory fitness and overall fitness level. Therefore, it is of great importance to improve adolescents’ overall health perception by improving their physical fitness level by concentrating on cardiorespiratory function other than improving flexibility and speed. The possible explanation is that people who get high scores in self-rated health tend to increase their perceived health through active exercise. Eriksen et al. (2013) further explained that Such self-motivated exercise formed a virtuous circle to encourage teenagers to keep fit. Jaakkola et al. (2019) also showed that teenagers who perform well in physical fitness tests are also full of confidence in their sports ability, which will further affect the social aspects of life achieved through sports activities.

In terms of emotional health conditions, the study measured the effects of two variables: depression and anxiety. Evidence showed that there was a negative relationship between the degree of depression and cardiorespiratory function. To be more specific, adolescents with poor cardiorespiratory function are more likely to score lower in their emotional functions. No significant correlation was discovered between the score of muscle strength and the level of depression in adolescents. This reflects the unique and important positive impact of cardiorespiratory function on teenagers’ emotional function. Correspondingly, the degree of anxiety and cardiorespiratory function are negatively correlated with each other. Adolescents with a higher level of anxiety are more likely to have poor cardiorespiratory health. Although a correlation was found between self-reported health and all health-related outcomes, the other three parameters did not show statistical significance with self-reported health. Nevertheless, despite the different voices, the majority of scholars recognized the positive correlation between overall physical fitness and emotional state. Many studies have been done to investigate the relationship between physical fitness and emotional wellbeing. For example, Harvey et al. (2018) believes that only 30 min of moderate exercise per week is related to a reduced risk of depression. However, some evidence indicated other factors that contributed more to mental health. For example, the data shown in this study is inconsistent with the results of Hallgren et al. (2020), who explored the relationship between cardiorespiratory health and mental health symptoms. This finding shows that it is exercise frequency, rather than cardiorespiratory factor, has a stronger correlation with mental health symptoms as the longer average weekly exercise time (lasting 1–2, 3–4, and 5–6 h, respectively) does not bring additional protection to the body. In addition, evidence also exists illustrating that only special forms of exercise, such as resistance training, have been proved to be effective in the treatment of emotional disorders and related symptoms (Gordon et al., 2018), but may not significantly improve cardiorespiratory levels. Besides physical exercise, the randomized controlled trial performed by Cunningham et al. (2021) revealed that both relaxation training and physical activities have buffering effects to reduce problematic emotions, indicating further research on different interventions to decide which one is more effective. In conclusion, cardiorespiratory health serves as an option to prevent common mental health symptoms, but necessarily the exclusive one.

Although the health measures adopted are self-evaluated, the results are consistent with other experimental research about physical fitness. Haverkamp et al. (2022) employed 361 adolescents to assess the relationship between their physical fitness and psychosocial health. The selected evaluation of physical and mental health is similar to this study: the evaluation of physical fitness includes muscle strength, speed and agility, as well as body mass index. Social mental health includes self-concept, depression, and anxiety. The results showed that adolescents with better cardiorespiratory function had higher self-concept and fewer depression and anxiety symptoms. In general, this evidence proves that cardiopulmonary function has an important impact on adolescents’ physical and mental health, which is not only reflected in self-perceived physical health but also reflected in mental health. The enhancement of muscle strength has a positive correlation with physical health but has little correlation with mental health. This indicates that schools and communities can pay more attention to the training of cardiopulmonary function and assist a certain degree of muscle strength training in enhancing the physical and mental state of adolescents. Some studies have proposed possible explanations for this result. Mücke et al. (2018) stated that young people with good cardiopulmonary and muscle functions will naturally participate in more sports activities. Sports have been proved to improve the level of endorphins in the brain and reduce the activity of sympathetic nerves, which can effectively reduce the psychological problems of teenagers (Nabkasorn et al., 2006; Shahr-Jerdy et al., 2012). In consequence, this study shed light on the importance of physical exercise to improve adolescents’ physical and mental health, especially cardiopulmonary training. The results also suggest that self-rated health and fitness level can be an important indicator to determine the health level of adolescents.

This study has had inherent limitations in its research design, measures, and participants. First of all, owing to the cross-sectional research design, the current study could not draw some causal conclusions; the directionality of the association between independent and outcomes could not be determined. Beyond that, this study utilized self-reported measures to collect data on all the variables, which were subject to recall bias and social desirability of the participants. Thirdly, as this study adopted a convenient and non-probabilistic sampling method, the research findings might be more regionally replicable than nationally replicable. Furthermore, the response rate was 63%, which was relatively low in the present study. Last but not least, although adolescents’ self-reported nature introduces several biases, the literature is consistent with self-reported validity and reliability; and self-reported use during the pandemic allowed continuing to assess physical fitness when it was impossible by objective methods. Given these limitations, future studies will be encouraged to address them to generate stronger evidence.

## Conclusion

This study offered some evidence concerning the associations between self-reported physical fitness in children and adolescents with some mental health-related outcomes, highlighting the roles of self-reported physical fitness in preventing mental health disorders.

## Data availability statement

The original contributions presented in this study are included in the article/supplementary material, further inquiries can be directed to the corresponding author.

## Ethics statement

The study procedure and protocol have been performed upon approval from the Institutional Review Board (IRB) of the Shanghai University of Sport (102772021RT071). Written informed consent to participate in this study was provided by the participants’ legal guardian/next of kin.

## Author contributions

CS and JY: writing – original draft. JY and LW: formal analysis. CS and HS: writing – review and editing as well as supervision. All authors contributed to the article and approved the submitted version.
